# The Hospital. Nursing Section

**Published:** 1906-10-27

**Authors:** 


### The Hospital.

### Nursing Section.

Contributions for "The Hospital," should be addressed to the Editor, "The Hospital"
Nursing Section, 28 & 29 Southampton Street, Strand, London, W.C.

No. 1,049.—Vol. XLI. SATURDAY, OCTOBER 27, 1906.

### Notes on News from the Nursing World.

### OUR CLOTHING DISTRIBUTION.

Perhaps because of the remarkable prolongation of the bright, mild weather, and the consequent inability of some to realise that Christmas is rapidly approaching, the contributions to our Clothing Distribution have not come in as we expected. We therefore remind our kind friends that we are relying upon them to help us to relieve at the Christmas season the necessities of some of the many thousands of sick poor of both sexes and all ages in the hospitals and infirmaries, who so urgently need to be supplied with warm garments. We acknowledge the receipt of a parcel from Miss Sharwood, of Hove. All parcels must be sent addressed to the Editor, 28 and 29 Southampton Street, Strand, London, W.C., and should have "Clothing Distribution" written on the outside.

### SISTERS AND SMALL STATION HOSPITALS.

We understand that, with the idea of cutting down the expenses of the War Office, no nursing sisters are in future to be permanently employed at small station hospitals containing fifty beds or under. In the event of a case being serious, the authorities are to apply to Millbank Hospital for a sister to nurse the patient. This means that members of the Army Nursing Service Reserve who are still on duty at the small hospitals will be replaced by sisters attached to Queen Alexandra's Imperial Military Nursing Service, in accordance, we learn, with the policy of the Army Medical Department. This policy is, we presume, dictated by a desire to promote economy, but the latter may easily be pushed too far. Wherever there is a danger of the sacrifice of efficiency, it becomes necessary to enter a protest. It appears that at some of the small hospitals the sisters' quarters have already been dismantled, and that there is now no accommodation available either inside or outside. We are afraid that in such conditions if a bad case arises there will be a temptation to try and save the inconvenience and trouble of calling in a temporary nurse by handing the patient over to orderlies. A system which has been justified by results should not be abandoned merely to ensure a slight reduction in the Army Estimates.

### EVICTIONS FROM THE NURSES' HOSTEL.

To the accounts of the annual meeting of shareholders in the Nurses' Hostel, Limited, last Thursday, supplied by well-informed correspondents, which appear on another page, there is not much to add. It will be seen that the directors of the Board decline to discuss the question of Miss Hulme's dismissal from the post of lady superintendent on the ground that an action in a court of law is impending. Our correspondents point out that "this was a convenient plea, and one to which it "was not easy to make a reply, though it points to "the conclusion that the Board have finally resolved "not to offer Miss Hulme any apology for the "course which has been pursued, unless or until it "becomes obligatory on their part. Unfortunately, from one cause or another only eight out "of the 170 nurse shareholders were able to attend "the meeting, and in the circumstances it was considered useless to demand a poll. The appointments of Miss Chamberlain as superintendent, "and Miss Kett as secretary were officially announced, and the Board appeared to think that "the shareholders ought to be fully satisfied with "the dividend they receive on their shares. "In fact, the latter were told at the meeting" that "if they were not satisfied, they could sell their shares." Since the meeting there has been another development which has materially increased the dissatisfaction of the nurse shareholders. On Friday morning Miss Wood for the second time said farewell to the nurses at the breakfast table, and urged them to report to headquarters if anything went wrong, instead of "washing their dirty linen in public." A little later in the day three of the nurses who are known to be friends of Miss Hulme received notice to quit in a month. It would thus appear that the Board purpose to follow up the inexplicable dismissal of a superintendent whose character and capacity they do not attempt to impugn, by evicting from the Hostel those tenants who have had the courage to ask for fair play on her behalf.

### PUTTING THE CLOCK BACK.

The National Poor-law Officers' Association, at their conference in Manchester, adopted the report of the Parliamentary Committee on the Nurses' Registration Bill, to which exception is taken on several grounds. Apart from the question of the principle of State registration, which the Association approve, we think that they have made a serious mistake on an issue of great importance. They condemn the Nurses' Registration Bill because it does not provide for the recognition of nurses who have only been trained for two years. As Mr. Percival pointed out, the Order of 1897 requires that a superintendent nurse must have had three years' training; and the recognition by any Central Nursing Board which may be formed, of

Poor-law infirmaries must, in the interests of nursing, be limited to those schools in which three years' training is given. The Association demand the appointment on the Central Board of a representative of this Association, and of the matron of a Poor-law infirmary. It is a pity that they have weakened their position by trying to include amongst acknowledged training schools institutions in which the standard laid down by the Order of 1897 is not adopted.

### AUCKLAND GUARDIANS AND THEIR SPECIAL NURSE.

A NURSE who was specially engaged at the Auckland Workhouse had her left hand injured by an insane patient whilst she was in attendance, and the medical officer informed her that an operation was necessary. Whereupon she went to the Guardians and asked them if they would pay the expense. The letter was discussed at their last meeting, but no decision was arrived at. A lady Guardian suggested that some idea of the cost might be furnished, which was not an unreasonable proposal. But it is reasonable on the part of the nurse to ask that she shall not be saddled with the cost of an operation rendered essential by an injury inflicted when she was on duty. We note that one of the Guardians urged "that it was not a case worth considering, especially as the nurse does not belong to us." If this view of the matter be adopted by the Auckland Guardians, we anticipate that when next they require a special nurse they will find it very difficult to obtain one.

### THE OWNERSHIP OF A NURSES' UNIFORM.

A CURIOUS question was raised at the last meeting of the Blean Guardians by the Chairman, who said that a nurse who was recently in their service had, on leaving, taken away the uniform supplied to her by them. He asked for an expression of opinion on the matter. The Clerk, we think, promptly justified the action of the nurse entirely by his statement that the nurses had always been allowed to regard the uniform as their property. But the Blean Guardians can, of course, make a rule to the contrary, or they can decide that the uniform is not to become the property of the nurse unless or until she has been in their employment for a specific period.

### EXONERATION OF A POOR-LAW NURSE.

THE Chorlton Guardians, having fully discussed the report of the Workhouse Committee into an investigation of the alleged ill-treatment of a patient by a nurse at Withington Workhouse, exonerating the nurse from all blame, have adopted the conclusion of the Committee. Dr. Rhodes, who took part in the discussion, affirmed that there was no ground whatever for the complaint against the nurse, and only three of the Guardians voted for a proposal to refer the report back. The latter threatened that the Local Government Board would be asked to hold an inquiry into the matter, but in the face of the determination arrived at by the Guardians, after a patient hearing of the case, we do not believe that the central authority will think it requisite to intervene.

### AN ADVERTISEMENT FOR GENTLEWOMEN.

AT the last meeting of the Darlington Town Council attention was called by the clerk to an advertisement issued by the Sanitary Committee for a nurse at the Isolation Hospital, which contained a condition that to fill the post "a gentlewoman was essential." The Town Clerk said that he sent out the advertisement, and that it was in the form that had been followed for many years. He himself had noticed the word "gentlewoman," and was doubtful whether to include it; but he stated that the matron wished it, "as it kept off a large number of undesirable candidates." Subsequently, however, he promised that the words should be deleted from the advertisement.

### THE IMPERIAL MILITARY NURSING SERVICE.

WE are officially informed that Miss E. R. Collins, Miss L. A. Ephgrave, Miss K. F. Fawcett, Miss C. C. M. Gibb, Miss M. Ironside, Miss A. C. M. Jameson, Miss C. H. MacCarthy, Miss F. E. Morton, and Miss G. H. Sellar have been appointed staff nurses in Queen Alexandra's Imperial Military Nursing Service.

### DEVELOPMENT OF THE NURSES' SOCIAL UNION.

THE Bath Centre of the Nurses' Social Union, though but newly formed, seems to have already struck root firmly. The third meeting was held this month by the invitation of Miss Stokes at her house in St. James's Square. An interesting lecture on "The Nursing of Typhoid" was given by Dr. Wilmot Herringham, of St. Bartholomew's Hospital, who travelled from London on purpose. It was a hopelessly wet day, but sixty nurses were present, and were generally of opinion that they were well rewarded, by their enjoyment of the lecture, for their enterprise. After tea the loan exhibition and some excellent music divided the honours until it was time to part. The heads of the nursing profession in Bath have taken the work of the Union up warmly, and there seems to be every prospect that it will really meet a need in the town. The Bath Centre is the one in which there are most nurses, the Minehead in which there are fewest, and in which weather is a most potent factor in attendance, as the district includes parts of Exmoor and places twelve miles away from the nearest station. Yet no fewer than fourteen were present at a meeting held in Minehead on October 18. Two nurses bicycled twenty miles in order to attend it. The lecture by Dr. Sanguinetti was on "The Uses of Alcohol in Medicine," and gave rise to considerable discussion, one nurse bearing off in triumph the text of it for future reference.

### CONFERENCE IN MELBOURNE.

A CONFERENCE between representatives of registered training schools and the Royal Victorian Trained Nurses' Association took place in the Melbourne Town Hall on September 6. There was a very encouraging attendance, and though it is extremely difficult to convince these gentlemen who are responsible for the management of the small country hospitals that a hospital under twenty beds makes an undesirable training school, there was nothing but friendly argument. Much of the work done by the Association was confirmed, and the rest postponed for a few months to enable another conference to be arranged.

### AN APPEAL FROM THE MOTHERS' UNION.

The President of the Mothers' Union, Mrs. Sumner, has addressed a letter to trained nurses. She appeals to them to join the organisation and to aid in its objects, as they have opportunity, particularly by seeking to arouse in mothers under their charge a sense of their sacred and tremendous responsibilities. A number of Queen's Nurses already belong to the Union, and Mrs. Russell, St. Stephen's Vicarage, Battersea Park, S.W., who acts as correspondent for nurses, will gladly supply any information desired.

### DEVONSHIRE AND PLAISTOW.

A great compliment has been paid to the managers of the Maternity and District Nurses' Home at Plaistow. At a meeting of the local branch of the Devon Nursing Association Lord Mount Edgcumbe proposed the establishment of a training home in the three towns—Plymouth, Devonport, and Stonehouse—on the model of that at Plaistow. The idea of Lord Mount Edgcumbe, and of other speakers who followed him, is that the training at a local home of the nurses required would be less expensive, and that by the establishment of such an institution the demand for them could be more expeditiously met. If the project is taken up and is successful on a small scale, it is probable that an attempt to train maternity nurses for the whole of Devonshire and Cornwall in the local home will be made.

### A QUEEN'S NURSE FOR HUNSTANTON.

It has been decided to form a District Nursing Association at Hunstanton. This movement is the outcome of the very admirable work done by a nurse who is leaving the parish. At an influential meeting held in the Town Hall the question of forming a branch of the Jubilee Institute, or of obtaining a nurse connected with the Norfolk Federation, was discussed. The feeling of the meeting was so entirely in favour of proceeding at once with the establishment of the Association that the alternative proposal to ascertain the wishes of the inhabitants of the town was set aside. It was stated that contributions from patients to the extent of £20 a year might be looked for, and there should be no difficulty in providing the remaining £80 which would cover the cost of a Queen's Nurse.

### A NEW AUSTRALIAN INSTITUTION.

Under the auspices of the Salvation Army in Australia an Intermediate Hospital has been opened in Melbourne. The building is detached, and well adapted for its present use. In front there is a garden, with large pepper-trees on three sides, and at the back ample space for laundry and other out-buildings. There is a large reception-room on the right and a room of similar size on the left, which does duty for bed-sitting-room for the matron. The dining-room is the only room occupied by the nursing staff, their quarters being in the home close by. The operating-room is on the ground floor. There is accommodation for forty patients, medical and surgical, and each patient may be attended by his (or her) own doctor. The charge made is from thirty shillings to three guineas per week inclusive. The diet is more varied and more daintily served than in a general hospital. Four medical men on the staff attend free of charge any patient unable to pay more than nursing expenses. There is no intention of training nurses for outside work; any help not required in the hospital will be used for Samaritan district work. But as the matron is a trained nurse she will probably recognise the advantage of educating her staff on the lines laid down by the Victorian and New South Wales Nursing Associations.

### THE PENSION FUND AND CAMBRIDGE NURSES.

An address on the aims and objects of the Royal National Pension Fund for Nurses was delivered by the Secretary, Mr. Louis H. M. Dick, on Wednesday of last week, at Addenbrooke's Hospital, Cambridge. There was a large attendance, and amongst those present were Mrs. Finch, Mrs. Huddleston, Mrs. Peart—members of the Committee of the hospital—and Miss Blomfield, the matron.

### IRISH MATRONS AND MASSAGE TEACHING.

It was decided at the first meeting of the Irish Matrons' Association, recently held, to arrange for the examination of the Incorporated Society of Trained Masseuses to be held again in Dublin, probably in February, 1907. Classes to prepare pupils have already commenced, and it is hoped that many candidates will present themselves for examination.

### FOR THE BENEFIT OF PUPIL MIDWIVES.

Owing to a considerable demand, the Committee of the Midwives Institute and Trained Nurses' Club, 12 Buckingham Street, Strand, W.C., whose medical library is so well known, have authorised the Secretary to allow pupils who are preparing for midwifery, massage, or sanitation examinations to use the books for three months for a small payment. This, of course, does not carry any rights as to the club, but only that books may be borrowed. Pupils must furnish a sufficient reference, and the arrangements are left in the hands of the Secretary who will report to the Committee early next year as to the success of the plan.

### SHORT ITEMS.

Princess Henry of Battenberg has promised to open a bazaar to be held in aid of the Maternity Charity and District Nurses' Home at the Canning Town Public Hall on December 13 and 14.—Under the will of Mrs. Cardwell, of Harrogate, the sum of £5,000 has been bequeathed to the Dewsbury District Nursing Association.—On Thursday, November 1, there will be unveiled, by Katharine, Duchess of Westminster, five stained-glass windows which a number of friends have placed in Chester Infirmary in memory of the late Frances Mary and Emily Wilbraham, two ladies who, in their day, rendered great service in assisting to nurse the sick poor.—There will be three lectures by Dr. Calder to certified midwives at the Midwives' Institute on the last three Tuesdays in November at 7 p.m., the subjects being "Obstructed and Delayed Labour" on the 13th and 20th, and "The Indications for the Use of the Various Drugs required in Labour and their Actions" on the 27th.

### The Nursing Outlook.

"From magnanimity, all fears above;
From nobler recompense, above applause,
Which owes to man's short outlook all its charm."

### A NURSES' HOSTEL AS IT MIGHT BE.

To fulfil its purpose a model nurses' hostel must provide the best possible accommodation of the most suitable character at an inclusive and reasonable cost. The whole establishment must be conceived and carried on from the standpoint of what the guests need and must have provided for them, including homelike comforts and the opportunity to enjoy, with their friends, reasonable companionship. Rational amusement of every kind should be encouraged. The regulations should be few, and as free from vexatious restrictions as possible. The whole atmosphere should be permeated by a genial spirit and exhibit an air of comfort and hospitality. The best-managed hotels, in this country, offer these advantages to their guests, and what a hotel can do for the public, the hostel ought certainly to be able to secure, to the fullest extent, for its nurse-residents.

When we come to consider the details of such an establishment it must be at once apparent that a management which does not provide the fullest facilities for ready inter-communication, between nurse-residents, practitioners and patients or their friends, is not entitled to be regarded as fulfilling, in any real sense, the true ideal of an efficient hostel for nurses. Nurses who undertake private work have necessarily to travel, and their times of arrival and departure must consequently be very irregular and uncertain. The ideal hostel must therefore be organised upon a plan which will provide that every guest shall meet with the fullest consideration and assistance, so as to lessen the difficulties she has to encounter in going and coming. The staff of a hostel for nurses must be carefully trained and exceptionally well organised. Every member of such a staff should be ready to cheerfully facilitate everything which is calculated to make the hostel a house of comfort to every guest who supports it with her patronage. The attainment of this object must necessarily entail some strain and self-denial upon the attendants, but an adequate training and careful administration should readily secure the accomplishment of this end without friction or difficulty of any kind.

Again, any hostel which is not organised to receive and care for a nurse who arrives by train in the early morning, which cannot provide her with a suitable meal and adequate accommodation at all times on her arrival, must be held to be inefficient and unbusinesslike in its arrangements. In the same way a member of the staff should have charge of the whole of the postal arrangements and messages, so that every communication may invariably reach the nurse-resident with promptness. The telephone arrangements should include enough boxes to enable several guests to use the telephone at the same time. A trained attendant should be charged with the entry in a suitable book of all messages received from doctors or patients. This attendant should see that if a nurse is out when the message arrives she shall receive it, and indeed every communication, immediately she returns to the hostel. The rooms should be large enough to enable a nurse to have at least one of her trunks or boxes with her, and the box-room accommodation should be ample, and so situated as to readily facilitate access to its contents, their removal and replacement. The manageress or superintendent should make it her business to study carefully the requirements and difficulties of the guests, upon whose goodwill and patronage the success of such a hostel must depend. In this way alone is it possible to ascertain and provide for numerous small matters which collectively make the difference between comfort and misery in the case of nurses who, as women-workers, have to be always ready to answer any call which may come either by day or night.

There are at the present time from 26,000 to 30,000 certificated trained nurses, of whom probably 25 per cent., or between 6,000 and 8,000 nurses are engaged in private work. The Nurses' Hostel during the twelve months ending September 30 last only had 1,027 visitors, a fact which may be taken to show that there is ample room for an ideal nurses' hostel in London of the most modern type, where nurses would find every comfort and facility, and be guaranteed that everything was so organised as to secure them the minimum of trouble and anxiety. An ideal hostel should be ideally planned, and there is no doubt that architects have not yet fully appreciated what such a building is capable of being made, and ought in fact to contain, in the way of accommodation.

We look forward to the time when London, and some of our largest provincial cities, will contain model hostels for nurses. In such case the lot of the private nurse will become much happier and easier than it is in present circumstances. The Nurses' Hostel has no doubt met a want which nurses have experienced, but the correspondence we have published proves to demonstration that there is need for reorganisation and amendment. We hope that the recent troubles may lead to great improvements, which should increase the Hostel's popularity. It might win for itself much greater support than it has ever yet attained.

### The Care and Nursing of the Insane.

By Percy J. Baily, M.B., C.M.Edin., Medical Superintendent of Hanwell Asylum.

6K

### II.—NURSING THE SICK.

(*Continued from page* 21.)

A most important item in the room is the fire-place; no sick-room should be without one, and a fire should be kept up during the night as well as during the day, except in hot weather. As we shall presently see, the use of the fire is not only to supply warmth and give an air of cheerfulness to the room, but it is also a very great aid to ventilation.

The bed is without exception the most important matter for which the nurse is responsible, and the manner in which it is kept is an indication either of good nursing or otherwise, for upon this to a very great degree depends the comfort or discomfort of the patient. The bedstead should be a narrow one, not more than three feet six inches or four feet wide. It should stand out in the room with the head against one wall, so that it may be easily approached by the nurse from either side. In a ward where there are several windows along a wall the beds ought if possible to be arranged between them. The space beneath the bed must be entirely unoccupied. The material of which the bedstead is made should be iron, and the mattress, stuffed evenly with horse-hair, should rest upon woven wire or chain springs. Only in one class of acute cases—fractures of the lower limbs—is a spring mattress not to be used. In the treatment of these injuries the bed ought to be firm and unyielding. A bedstead provided with a woven wire mattress, such as the Lawson-Tait bed, may be rendered firm by the use of "fracture boards." These consist of ordinary deal planks in which several holes have been bored for ventilation, they should be a little longer than the width of the bedstead, and should be placed across the bed between the wire mattress and the hair one. They should, if possible, rest upon the iron frame of the bedstead. When the patient is suffering from some chronic condition which entails confinement to bed for a very prolonged period, especially when there is great emaciation or any form of paralysis, it may be necessary to use a water-bed in order to diminish the risk of the formation of bed-sores. There are many forms of water-bed to be procured, but probably the one which answers the purpose best is the old-fashioned tank variety, which consists of a metal tank about a foot deep which is partially filled with water. Over this is loosely spread a stout sheet of mackintosh, which is fixed to the wooden sides of the bed. In such beds the water will keep perfectly sweet apparently for an almost indefinite time.

The amount of bed-clothes necessarily depends upon the individual requirements of the patient. They should be light, and of sufficient thickness to prevent cold. The sheets should be of cotton twill—not linen—and beneath the under sheet there should be one or two folds of blanket. In cases of acute rheumatism (rheumatic fever), the patient may be allowed to sleep between the blankets. The under sheet is the principal part of the bed, and needs the constant care and attention of the nurse to prevent its getting thrown into wrinkles and folds under the patient. These and bread-crumbs in the bed are a source of very great irritation and discomfort to the patient, and are an important factor in the production of bed-sores. The under sheet should always be kept tightly stretched over the mattress, the overhanging sides and ends being carefully tucked in beneath it. Additional security may be obtained by attaching the sheet to the sides of the mattress with a few safety pins. When a bolster is used it must never be rolled up in the end of the under sheet, but must have a separate case. This and the pillow should be well stuffed with feathers. Like the sheet, the bolster and pillow cases must be made of cotton twill, and not linen.

In all cases where the patient's habits are faulty the sheets must be changed as soon as they become soiled, no matter how often this may occur during the day or night. A patient who is allowed to remain in a wetted bed runs great risk of becoming bed-sore, and the nurse who knowingly leaves her patient in such a condition deserves instant dismissal for utter incompetence. These cases should always be provided with a draw-sheet, which consists of an ordinary sheet folded into three or four thicknesses and laid across the centre of the bed beneath the patient's buttocks. The ends of the draw-sheet which overhang the sides of the bed must be carefully tucked in under the mattress and then pinned to it in the same way as the under sheet, so as to keep it taut and smooth. When the draw-sheet becomes wetted or soiled it can be changed with much less trouble both to the patient and nurse than the other sheets. In dealing with patients who are habitually wet a mackintosh sheet may be used to protect the mattress. Such a sheet, however, should not be used unless absolutely necessary, for it greatly interferes with ventilation, and prevents the evaporation of the moisture from the patient's skin, which is therefore apt to become sodden and softened—a condition which induces bed-sores.

### *Re-making the Bed.*

If the patient be not too weak, in chronic cases he may be lifted out of the bed while it is being re-made. While he is out of the bed he must, of course, be well covered to prevent chill. In more acute conditions, or where the patient is too ill to be treated thus, a second bed may be placed beside the first, and the patient lifted into it. If the patient is too ill or too weak to be dealt with in either of these ways the bed must be re-made by halves. To change the under sheet it must first be loosened and unpinned from the mattress all round and withdrawn from beneath the bolster, the patient must then be lifted on to one side of the bed and half the sheet rolled up and tucked against the side of his body. The clean sheet is then similarly half rolled up lengthways, leaving one-half unrolled. The unrolled part is then to be spread over the lower blanket, the rolled up part being made to lie close to that of the sheet which is being removed. The patient is then lifted on to the clean sheet, the soiled one removed, and the clean one spread out over the remaining part of the bed and arranged.

The draw-sheet may be changed in a similar way, or the clean one may be pinned to the dirty one, and after the patient, if strong enough, has raised himself, or if he be too weak to do this, has been raised by an assistant nurse, the soiled draw-sheet may be pulled away and the clean one pulled in under him. If this method be adopted, care must be taken to turn the end of the clean draw-sheet over before it is pinned to the dirty one, otherwise there is a risk of the safety pins scratching the patient.

To change the upper sheet all the upper bed-clothes except the sheet and one blanket may be removed and the clean sheet spread out over the blanket. The rest of the bed-clothes are then to be replaced over the clean sheet. After a few minutes, when the clean sheet has had time to be warmed, the soiled sheet and blanket are to be pulled out in the following manner:—See that they are all thoroughly free all round, then the nurse at the head of the bed holds by each corner all the bed-clothes except the sheet which is to be removed and the blanket immediately above it. These are similarly held by an assistant at the foot of the bed, and are then by her pulled out, leaving the clean sheet in its proper position. The blanket is then replaced on the bed beneath the coverlet and all the bed-clothes arranged and tucked in.

Instead of lifting off the upper portion of the bed-clothes the clean sheet may be placed in position in the following manner:—All the upper bed-clothes are to be thoroughly loosened from the mattress all round, and are then rolled up a little at the foot; a blanket which has been previously warmed is then to be passed into the bed between the patient and the upper sheet. To accomplish this the nurse and her assistant stand on either side of the bed at the foot, and, holding a corner of the warmed blanket in the hand of that side which is towards the patient's head, pass it beneath the bed-clothes and draw it up over the patient, while with the other hand the bed-clothes are retained in position. When it has been drawn in as far as possible in this manner the nurse goes to the head of the bed, and, leaning over the patient, completes the drawing up of the blanket while the assistant at the foot holds the rest of the bed-clothes in position. In exactly the same way the clean sheet is to be passed in between the blanket and the soiled upper sheet, which is then to be pulled out from the foot of the bed. After a short time, when all fear of chilling by the clean sheet has passed, the blanket is also to be pulled out. Instead of passing the blanket and the clean sheet in separately as described above, they may be laid one upon the other and both passed in at the same time. Before commencing to re-make the bed it is, of course, most essential to see that everything is ready at hand, and that the clean sheets have been thoroughly aired.

The patient's body clothing should consist of a gown made of flannel or some woollen material. If it be of cotton a light vest of merino or some similar material should be worn beneath it. Woollen materials readily absorb the moisture from the patient's skin, and therefore diminish the risk of chill when perspiration is profuse. Flannelette, which is a cotton fabric, is, however, fairly absorbent, and a gown of this material is both comfortable and cheap. In many painful conditions the task of removing the patient's nightgowns is a difficult one for the nurse and a trying ordeal for the patient. In such cases its removal will be much simplified if it be open through its whole extent in front and fastened with tapes. The sleeve also should be ripped up from the neck to the wrists and fastened with tapes.

*(To be continued.)*

### The Nurses' Clinic.

### LARYNGOTOMY.

As laryngotomy is advisable in case of any sudden obstruction of the larynx, and as no other operation calls for more care and vigilance on the part of the nurse, we should be careful to keep our minds fresh in every detail of this branch of nursing. I myself was trained in a large provincial hospital and medical school where the nurses were required to do two years' private nursing on the completion of their training. My first private case was to take night duty in a case of tracheotomy in a private house. The patient, a gentleman aged seventy-five of very nervous disposition, had been operated on about midday. I knew there was another nurse in attendance from another institution, and my mind was more or less relieved; but when I arrived she informed me that, although she had been eight years nursing, she had never seen a case of tracheotomy before, nor a case of diphtheria. How unfortunate for the patient if we both had been equally ignorant of this most important branch of nursing.

In such cases the surgeon usually brings everything required for the operation, and the nurse has only got to prepare the room as for any minor operation, remembering to have a small hard pillow to put under the patient's neck to keep it on the stretch. It might be well also for her to know the instruments required—namely, scalpels, forceps, tenaculum, blunt hooks, tracheotomy tubes of different lengths and sizes, dilators, ligatures and sponges, two pieces of narrow tape to fasten the tube round the neck. It is very important also for the nurse to be alive to the danger of infection.

Most surgeons fasten a silk handkerchief across their mouths in order to prevent their own air passages becoming infected with the poisonous material coughed up. This would look rather out of keeping on the part of a nurse, but she can be careful to wash out her mouth with some disinfectant when she has been in very close attendance on the patient for any considerable length of time. The air of the room should be kept pure and at an even temperature of 65°. The bed should not be placed near a hot fire, neither should the spray from the kettle be kept constantly playing over the patient's face and body. A sponge warm and moist should be always kept over the tube. The inner tube should be changed every hour when the discharges are thick. A great deal of harm may be done by constantly introducing a feather to clear the tube. If actual obstruction takes place it is better for the nurse not to attempt to clear it in this way, but inform the doctor at once. Most surgeons will give the nurse instructions in such cases to remove the tube altogether and gently introduce the dilators, keeping them

THE NURSES' CLINIC—*Continued.*

in till help comes. The patient will usually breathe quietly, except in the case of very young children; it is difficult to keep them quiet while the dilators are being introduced.

When the diphtheritic membrane is present, the use of a solution of perchloride of mercury—1 in 1,000—by means of a hand spray is recommended by some doctors, and is said to be more beneficial than solutions of soda bicarbonate or borax. In using the hand spray a tongue depressor will be required in order to let the fluid from the spray reach the pharynx. The patient usually finds it very difficult to talk or make known what he wants; therefore it will be well to supply him with a small slate and pencil, and let him write down what he wishes, except in the case of very small children. In no case must they be allowed to try to talk.

The patients, of whatever age, must be carefully fed in small quantities at a time and always with fluids, such as milk, beef tea, bovril, chicken soup (strained), etc. These must be given very slowly, as the least irritation may set up a fit of coughing.

### Incidents in a Nurse's Life.

### A CASE OF PUERPERAL MANIA.

I was staff nurse in a woman's special ward of six beds. It was 8 P.M., prayers had been said and everyone comfortably settled for the night, when a message was brought up, saying we were to expect a "probable immediate," "septic abortion." All beds were full, but we were able to transfer a patient, who was going out the next day, to another ward for the night. We then made up the bed and helped sister to prepare for the operation. The patient, a pretty little woman, arrived just before 9 P.M. The surgeon soon came and, after consulting for some time, decided not to operate just then. However, it had to be done the following noon, and everything went off satisfactorily. The patient brightened up considerably towards evening, but during the night her temperature went up, and kept steadily rising, so in the morning the doctor decided to inject anti-streptococcus serum 10 c.c. every four hours.

She was very quiet all day and lay with closed eyes, looking very exhausted and apparently taking no notice of anything. Four P.M. came round, patients' tea-time. My probationer, having prepared their teas, had gone down to have her own tea. I was alone.

The injection of serum being then due, I proceeded to get all in readiness for the doctor, who was expected in a few minutes. Having collected everything but the syringe, I put the screen round the bed, so that the other patients should not see what was happening, and went to get the syringe from the theatre, which was immediately opposite the ward door, across a very narrow corridor. I was not away a second, having gone into the theatre, taken the syringe off the table and out again, when on reaching the ward door, I saw an apparition that nearly froze my blood. The windows were not very high, and being a very warm day all were open at the bottom, and there was the patient more than half-way through the open window; part of one leg and the foot, the arm and hand of the same side, were the only parts visible by the time I reached the window, for she had seen me rushing toward her and hastened to evade me. I hardly dared stop to breathe until I clutched her leg with a firm grip, and pulled her in on to the window-sill. Then sister, who was off duty, but fortunately in her side-room, came upon the scene and helped me lift her back to bed. She had been attracted by the screams of the other patients, who had not noticed what had happened until they saw me rush across the ward; partly, I suppose, on account of the screen being at the foot of the bed, and partly because they were so busy having tea, with their backs toward her bed.

In less than five minutes from the time that she had first jumped out of bed the patient was tucked tightly back into bed, but what a five minutes it had been. She had apparently been feigning sleep and watching her opportunity, for she had the whole width of the ward to cross before reaching a window.

After this she was not left a second. On being asked why she did it she remarked that her eight children had been killed and she wanted to be with them. Of course, they are all alive; and when, a week afterwards, she began slowly to mend and her reason came gradually back, she told me how she had dreamed that she tried to throw herself through the window. We allowed her to continue to think it a bad dream. Although she was one of the dearest little women I have ever helped to nurse, yet I shall never forget the relief we all felt when she went home. She was quite cured, although for some time we despaired of both life and reason. I sometimes shudder when I think of what might have happened if I had delayed a second, for the ward was situated at the top of a five-story building.

### The Private Nurse and the Patient's Friends.

By a Correspondent.

There is no field which contains so many new experiences and such great possibilities as private nursing, and it is a thousand pities, although unhappily a fact, that the private nurse is not a favourite with the general public. Why this should be I do not know; but one thing I am sure of—that every nurse starting off to a private case should feel and make it her bounden duty to do all she can to alter the public's opinion of her profession. Of her duty towards her patient I need not speak; if she is worth her name she will need no instruction on that point. But the nurse should make it her duty also to please her patient's friends as well as to please the patient himself. This will have a salutary effect on the invalid and—a matter of not a little importance —it will make things so much easier and smoother for yourself.

There is a popular idea to the effect that the trained nurse is not at all "*timide*," that she is too apt to be overbold and self-confident. Now we know from personal experience that a great many women going to a new case suffer acutely from shyness at the mere idea of entering a household of complete strangers, and often hostile strangers, too. It is not the patient we dread to encounter. He is ill, poor soul, and requires the care and skill which we, with our long training and experience in sickness, can give him. In that direction we feel confident and assured. But the patient's friends! The wife, who, naturally enough perhaps, looks upon the nurse as a bold usurper of her rights and privileges; the daughters who have attended some fashionable "first-aid" lectures, and who feel themselves competent with a clinical thermometer! Remember that these people, however high in the social scale, are very ignorant on the subject of "nurses," and that they will, with characteristic impartiality, make a right or wrong estimate of the nursing profession according to the opinion they form of the nurse. My advice is, conciliate them. Be slow to take offence. Don't keep all the sick-room news to yourself. Tell as much as you can to the friends. Let the anxious wife feel that you know the patient would rather have her to wait upon him were it not too arduous a task for her strength. Consult her about little things. Ask her opinion occasionally, even if you do not make use of it. Don't let it "put you out" when the patient's friends insist on carrying off the temperature chart to the drawing-room! The patient himself is either too ill to care about such trivialities or else you may be tolerably certain he is vastly proud of that zigzagged sheet of paper of which he is the subject, predicate, and object! Therefore there is really no need for you to worry. You can even keep a private copy in a drawer while you leave one out for the friends to play with. Now this is really quite a common trick with the "lay" friends, but I have known it again and again to be the source of a vast deal of unpleasantness between the nurse and her clients.

When a little *contretemps* arises try to forget yourself. Imagine yourself in the place of these people; try to see yourself with their eyes. As far as in you lies, banish your "ego" for the time being. I cannot too strongly emphasise this point. If every young girl starting private nursing this autumn would take for her watchword "Self-forgetfulness," I am willing to prophesy that it would not take many years for the public to change its opinion of the much maligned "trained nurse."

### The Hostel of My Dream.

BY A PRIVATE NURSE.

Weary after a long journey and a heavy case, I arrived late one evening at a well-known abiding place for nurses. I had written a week before for a bed, but none had been reserved, so, as the place was full I was informed that I could have a camp-bed in the cloak-room or should be obliged to go elsewhere. The hour was late and I was worn out, so I took the cloak-room with a bed made up close to the hot-water pipes, just over the boiler room, and a big window with no attempt at a curtain behind my head. I got undressed and lay me down with a sigh, stretching my cramped limbs out on my mattress, which was hardly a bed of roses. I felt too tired even to sleep, and so just closed my eyes and thought to myself how differently I would arrange things had I the ordering of a place like this. Having arrived too late for supper and been told that no food was served between meals, I had gone to bed on a glass of milk and a piece of roll from the dairy near by—the neighbouring restaurants and tea-shops being closed. I thought that first and foremost I would have a restaurant so that nurses coming from or going to cases could have a meal when they wished it and thus bring in a profit which must amount to many pounds in the year and which was now spent in neighbouring shops. If a nurse wished for her breakfast in bed it should be served, at any rate decently, with a tray cloth and each her own tea pot, instead of a cup and saucer, both full of tea or coffee; the sugar should be in a basin and not simply two pieces chasing each other round the tray. Having a restaurant the nurse could have her breakfast at any hour convenient to herself in reason. When she got up she could go to the reading and writing room, where silence should be the rule, not the exception, and where she could either write to her friends and employers or read the papers in peace and quietness away from the gossip of the sitting-room. Later on some friends might call and she would take them into a club sitting-room for friends and outside members of the profession who desire a meeting place. This room could be used for lectures in the winter evenings, and members of the medical and nursing professions, as well as those interested in women's work generally, would be invited to give their opinions and experience. This would be largely for the benefit of the private nurse, who, as all know, is so tied to her headquarters when once she is booked for work, that she cannot go to outside lectures, meetings, or classes, thus making her life even more narrow and monotonous than it need be. Notices of impending lectures and meetings to be posted in all the public rooms in the building. The outside membership, as indeed all others, should be fixed at a fee within reach of most nurses, even those in training, payable if need be in two sums in the year. For all money spent in this institution the members, in and outdoor, should receive a coupon receipt, these coupons to be saved and produced when required for the making up of accounts, when a percentage would be paid on them, thus making each nurse a member of a co-operative concern in the truest sense of the word. All members must, of course, pay an entrance fee and should be encouraged to contribute to an old-age or retiring pension or home. An inclusive fee for those staying for a week or more could be charged either for cubicles or single room boarders for board and residence, and both these places should be sufficiently large to admit of at least one box for the comfort of members. Cupboards should also be provided, so that each member in or out could hire one if she wished. These could be placed in all corridors and stock rooms. Each cubicle, as well as each private room, should have a fireplace, window, and a hanging cupboard. A really competent manageress or housekeeper would preside, as well as a well-trained organising secretary, both of whom should be women of heart, as well as of brain.

I turn at the delightful thought and open my eyes, when, with a start I find that it is already daylight and I have been dreaming! What a disappointment! But why should it remain only a dream? Is it all quite outside the bounds of possibility? Are there no people in the world who would like to help our hard-working sisters to a happier state of things in their private life? If Rowton Houses, Peabody Buildings, and other such places can pay at the rate they do on the money invested in them; if the Howard de Walden Home paid off its debts and made a profit in a year from its restaurant only when it was once well managed; and if the Nurses' Hostel paid off the debt on the new building in two years, and pays its shareholders 4 per cent. without any restaurant, surely this little plan of mine would not be a bad investment for anybody's money.

### Midwifery Training.

BREACH OF CONTRACT CASE.

A case for breach of contract brought by Nurse Jukes against Miss Heatley, matron of the St. Clement's Maternity Home and Nursing Institution at Fulham, was tried before Judge Selfe at the Brompton County Court on October 16 and 19.

The undisputed facts of the case appear to have been that Nurse Jukes, who was a resident in Natal and had received six months' training at the Addington Hospital, Durban, came to England in the early part of this year in order to qualify as a midwife, so as to open a nursing home in Durban on her return. Having seen an advertisement of the St. Clement's Home in The Hospital she applied for a prospectus, and received one stating that the fees for a trained nurse for three months were £15 15s., and for an untrained nurse £25 for four months. Nurse Jukes replied that she wished to go in for the examination of the Central Midwives Board on October 25 (to pass which she would have to deposit certificates showing that 20 midwifery and 20 nursing cases had been attended three weeks beforehand), and would therefore enter the Home on June 25. Miss Heatley wrote on May 15 that as Nurse Jukes had only had six months' training she advised that she should take the four months' course. Nurse Jukes entered the Home on June 25, and on June 29 paid £15 15s., having previously paid £1 1s. entrance fee, and received a receipt from Miss Heatley for three months' midwifery training. At the end of a month Nurse Jukes complained that she was not getting her full number of deliveries. She had only had four deliveries and five cases in which the infant was born before her arrival, and had nursed five cases for ten days, and was then nursing her second batch of five. The three other nurses had, in nearly the same time, had respectively seventeen cases, twelve, and nine. According to Nurse Jukes' account, Miss Heatley told her that she should not give her any more cases until she paid the balance of the £25. Miss Heatley, however, said that she told Nurse Jukes that she should not give her her full number of cases till she paid the balance of £25, which, as Miss Heatley affirmed, she had promised to do when she received some more money early in September. Nurse Jukes left the Home on July 25, and there was conflicting testimony as to the reasons of her leaving, Nurse Jukes saying that the matron told her to go and Miss Heatley affirming that Nurse Jukes herself announced her intention of leaving. Before she left, Miss Heatley offered to return her £10 of her fees, but she refused to take it.

For the defence it was stated that attempts had been made of late to raise the standard of midwifery, the Central Midwives Board requiring a minimum of three months' training from a fully-trained nurse; that Miss Heatley would not undertake to train any nurse with less than a year's training (fever training counting the same as general) under four months; that at an interview on May 24 Nurse Jukes agreed to come for four months (this plaintiff denied); that on June 29 when plaintiff paid £15 15s. defendant gave her clearly to understand that this sum did not entitle her to the training of the Central Board; that plaintiff said she could not pay more at present, but would do so early in September (this was also denied by plaintiff); that plaintiff afterwards stated to the other nurses that she only meant to stay three months, as she saw there were plenty of cases for her to get them in in that time; and that when plaintiff complained that she was not getting enough cases defendant told her the nurses who were taking the three months' course must come first.

The case occupied some time in hearing, witnesses appearing for both sides. The Judge, in summing up, said that the whole matter resolved itself into whether there was a breach of contract or not, and in his opinion there was. He therefore gave judgment for the plaintiff for the full amount of the claim, £16 16s. and costs.

### Presentations.

Doncaster and Mexborough Joint Hospital.—Miss Edith Jackson, late matron of the Doncaster and Mexborough Joint Hospital, who was married to A. W. Frew, L.R.C.P.&S.(Edin.), at Mexborough last week, was previous to leaving presented with a silver cake-basket and a set of afternoon teaspoons as a token of the esteem of the staff.

### Everybody's Opinion.

**[Correspondence on all subjects is invited, but we cannot in any way be responsible for the opinions expressed by our correspondents. No communication can be entertained if the name and address of the correspondent are not given as a guarantee of good faith, but not necessarily for publication. All correspondents should write on one side of the paper only.]**

### THE NURSES' HOSTEL.

"Disgusted" writes: I should be very grateful if you could find space to insert this letter. As a shareholder in the Nurses' Hostel Company I attended the meeting on the 18th inst. in the hope that some explanation might be given as to the extraordinary treatment of Miss Hulme by the directors. An inaccurate and misleading statement was printed in the report, and no explanation was given. One of the directors seemed chiefly anxious to impress upon the nurses present the excellent dividend, and it was evident that the 4 per cent. ranked much higher in the Board's estimation than truth and justice.

"B. K." writes: The numerous friends of Miss Hulme are disappointed, but not disheartened, at the result of the annual meeting of shareholders, which was held at the Nurses' Hostel on October 18th. The turn matters have taken so far recalls the title of a book I once read, "Crushed yet Conquering." As we are contending for what is right against what is obviously and indisputably wrong, it must follow by all the laws of reason and morality that right must triumph. The members of the Board present were General Sir Allen Johnson (chairman), Miss Wood (managing director), Miss Paul (former secretary), Mr. Lionel Earle, and Miss Cooper. After the minutes of the last meeting had been read by the retiring secretary, the chairman made the usual statement concerning the management of the Hostel and the payment of dividends, etc. He then asked the shareholders present if they were satisfied with the report, whereupon Miss Stuart rose and affirmed that it was not correct, on the ground that there was a serious omission of facts. She went on to explain that the report spoke of Miss Wood as being superintendent until October 20th, ignoring utterly Miss Hulme's official position as such for the space of three weeks. Sir Allen Johnson gave a reply which was no explanation—namely, that the directors did not think it was necessary to mention that fact. Unfortunately he was not reminded that the omission was an illegality! Miss Stuart further objected that £25 of the shareholders' money offered to Miss Hulme in lieu of proper notice was an improper use to make of it. Many references were made and questions put by the shareholders concerning the dismissal of Miss Hulme. The questions were evaded by the members of the Board, who sheltered themselves by saying it was a matter for her solicitor, as an action was impending. It was very evident to those present that the mind of the Board was uneasy on account of a rumour that had reached them that another hostel is about to be started. If this is the case, it must be the direct result of the shortsighted policy of the Board, who seem just now to be warring with their own interests. The subject of Miss Hulme's dismissal is of far more importance than may appear at first sight to those not deeply interested. In contending for her vindication we contend for the vindication of our whole profession, so that it is imperative Miss Hulme's good name should be cleared. I should like to draw the attention of "One of Miss Wood's Admirers" to the fact that in 24 hours 47 nurses had signed the letter addressed to General Sir Allen Johnson, protesting against the unlawful dismissal of Miss Hulme; in all 57 names were appended in less than two days. Had there been more time, twice or thrice that number would have signed. The petition was another thing, and a larger number signed that. Six hundred nurses cannot reside at the Hostel at the same time, seeing that each block is only built for 50.

"An Indignant One" writes: I hear that a great deal of indignation has been caused at the shareholders' meeting of the Nurses' Hostel Co., Ltd., by the speech of one of the directors. In replying to a nurse who complained of the discourtesy she experienced when residing there the director said that she had stayed from time to time at the hostel and was always well treated there, and considered that the inmates were very liberally supplied and at a low cost. In fact, she continued, "She overhead two nurses talking together, one of whom said to the other, 'You can't possibly live at the hostel for 17s. 6d. per week. Surely it must be a charity.'" Just imagine that being allowed to pass with only a single protest from a body of women, many of whom were in residence at the time. It is quite a hopeless task to help them. When they come to a point where they can redress their wrongs they have not the courage to support their own cause, though inwardly furious and complaining amongst themselves. They deserve all they get. No wonder they are treated with contempt. The lady who narrated the overheard conversation must have been sadly deficient of a sense of humour, or she would have recognised that the nurse spoke ironically about the "charity." That might account for people being treated as paupers, but not like inmates of a reformatory. Allow me to add that the "charity" pays over £600 per annum in salaries and servants' wages—a unique "charity" I must say, where the recipients have to pay a penny more for each telephone message received than they would have to pay anywhere in the United Kingdom."

"A Victim" writes: There appears to be an idea that the petition presented at the Nurses' Hostel was signed by newcomers; this was in no way the case, the majority being the names of those who had been in the habit of coming to the Hostel for two, four, and eight years, many since its first opening. It was talked of long before Miss Hulme's advent, whatever Miss Wood may have been led to think to the contrary, and many signatures obtained. That there were not more at the end was due to the fact that it was impossible for all names to be known to the originators, or for them to be traced, besides which more than a few would be of the "peace-at-any-price" party. In consideration of Miss Wood's age it was not presented long before, and it was hoped that with new blood and more modern ideas we should get quietly and privately what we desired. However, Miss Wood's behaviour to Miss Hulme forced our hand, and we nurses, who have meekly borne slight after slight for years, rose up in a body and demanded a reason for such conduct. Three times we asked it, and as no answer was vouchsafed, we filed from the dining-room, leaving the managing director face to face with Miss Hulme, to say her grace after meat as best she could. After this no other course than the one taken could be pursued. Small concessions were given in the hope of stemming the tide, and every effort made to throw the whole blame on Miss Hulme, with the result which we have seen.

### A TEARING RAGING CRITIC.

"A Lover of Truth" writes: Having known Miss C. J. Wood for over seventeen years, I am naturally indignant at the manner in which she is being treated. I have made the Hostel my home for several years now, and have always been most happy there. But some people are never satisfied; they are "chronic grumblers," discontent possesses them, and they would like to rule the place. Others conceal their passion until they see some advantage to let it out. When the restraint is taken away, people, as we say, show themselves in their pure naturals; unloose a tiger or a lion and you know what he is. A malicious heart and a slanderous tongue go together; they do nothing but plot against the just; they are plotters and ploughers of mischief; they are skilful and industrious in it. If their malice have not a vent in hurting some way they will burst for anger. We may see by their language what their heart saith, and what their tongues would vent if they dared. Some people, disliking serious and settled thoughts, entertain everything as it is offered them at first, and suffer their imaginations to rest therein without further judging. Thus it is that the best things and persons suffer much in the world. We complain of the times; but let us take heed we be not a part of the misery of the times, that they be not worse for us. Everyone who knows Miss Wood at all knows that she is a noble woman, a woman of sound spiritual judgment, self-denying, and devoted to good works, whose care has been to build her profession not on seeming appearances, but upon sound ground, so sound that the "gates of hell cannot prevail against it." It is not in our power what other people think or speak, but it is in our power, by God's grace, to live so that none can speak ill of us but by slandering, and none believe ill but by too much credulity; and I am sure that by using a little spiritual eye-salve anyone could easily see that this is the kind of life Miss Wood lives. She is certainly not a woman that would delight to be blown up with flattery, neither should I think her greatest care is how to compose her outward carriage in a graceful manner, but she certainly must have studied how to compose her spirit. It would be well for the world if there were more women like her. What ingratitude it is that her last few days at the Hostel should be tinged with so much contention! But there is a counsel in heaven that will easily destroy all contrary counsels on earth; and however much it may vex those that rally round the enemy, I do sincerely hope they will see at length a disappointing of their projects.

### THE ACTION AGAINST THE MATRON OF A MATERNITY HOME.

Miss Heatley, Matron of St. Clement's Maternity Home and Nursing Institution, Fulham, writes under date of October 20th: In case that you consider the action decided yesterday in the Brompton County Court brought by Mrs. M. Jukes against myself is of sufficient public importance to be reported in your paper, I wish to bring to your notice one important fact. My reason for contesting Mrs. Jukes's claim was not primarily a question of fees or money at all, but that I wished to make it clear publicly that I do not undertake at St. Clement's Maternity Home and Nursing Institution to train pupils for the Central Midwives Board examination in less than four months, unless they are already trained nurses. By a trained nurse I mean one who has had at least twelve months' general training in a hospital. I consider that no untrained nurse can properly qualify herself for the examination in less than four months. I have proved this by practical experience, and as a consequence I do not accept untrained pupils for a less period. If the result of my case has made this fact clear, I shall not regret the time and money spent in defending the action.

"One of the Pupils" writes: I am at present a pupil midwife in St. Clement's Home, Fulham. My experience has been very different from that stated in court during the trial of the action against the Matron. I have had many years' nursing experience, as probationer, nurse, sister, and matron, in large hospitals at home and abroad. This is a well-conducted home and a good training school for midwifery, and the fees for the training are by no means excessive. There is plenty of work for the pupils, and instead of the twenty cases demanded by the Central Midwives Board they get from thirty to forty.

### THE DIFFICULTIES OF THE PRACTISING MIDWIFE.

"Another Midwife Who Loves Her Work" writes: I have read with much interest the letters from one who loves midwifery cases, and I fail to see how any conscientious midwife with tact and perseverance is unable to make a living. I have been in private practice for eleven years and have got a connection together of over 200 cases yearly. I can make a good living, besides being able to do a little charity work where it is necessary. I have my own home to keep going and pay a maid a fair wage, and am able at times to put away for old age. I have always found the medical men willing to come at once to render me any assistance, and I have had the greatest kindness and courtesy shown me by them. In fact, I have been often sent for to assist when a nurse has been needed with any of their poor cases. Of course, there are some midwives I know who cannot get on with the medical men, but I think that they are partly to blame. The rules of the Central Midwives Board are certainly very strict, but if we are unable to adhere to them I am perfectly sure that the Gamp cannot. I do not think that any trained midwife who does her work well need fear. If we all gave up the work the poor mothers would be left entirely to the mercy of the Gamp whom I hope will be soon abolished altogether. Then, the nurse midwife will be a boon to many. I might just say by way of encouragement to others that I have attended nearly 3,000 cases and never had a fatal one.

### Appointments.

Bromsgrove Isolation Hospital.—Miss Laura Charles has been appointed staff nurse. She was trained at Carnarvonshire and Anglesey Infirmary, Bangor, and has since been staff nurse at the Borough Hospital, Eastbourne. She has subsequently done private nursing.

Camberwell Infirmary, Brunswick Square, S.E.—Miss G. M. Bailey has been appointed sister. She was trained at the Royal National Hospital, Ventnor, Isle of Wight, and Chelsea Poor-law Infirmary. She has since been sister at Kingston Infirmary, Kingston-on-Thames, and sister at St. Pancras Infirmary, Pancras Road, N.W. She has also done private nursing.

Crewkerne Cottage Hospital.—Miss Mabel A. Lloyd has been appointed matron. She was trained at the Royal Infirmary, Edinburgh. She has since been sister at Grantham Hospital; sister at Halifax Infirmary; sister in charge of Male Surgical Home in Harley Street, London; and matron at Walmsley Clergy Home of Rest.

Hospital for Special Diseases, Cambridge University. Miss Mabel R. Smythe has been appointed matron. She was trained at Charing Cross Hospital, London, and was subsequently in charge of the Electrical Department for a year, and also of the Light Department for six months. She has also done private nursing.

Inverurie Joint Epidemic Hospital.—Miss Mary Bryce has been appointed matron. She was trained at Edinburgh City Hospital for fever training and at Dundee Royal Infirmary for general training. She has since been ward sister at the latter institution.

Isolation Hospital, Menston in Wharfedale.—Miss R. Ives has been appointed sister. She was trained at Portsmouth Infirmary, where she has since been sister. She has also been staff nurse at Leeds City Hospital, and charge nurse at the Grove Hospital, Tooting, S.W.

Ladies' Charity and Lying-in Hospital, Liverpool.—Miss Edith Kirk has been appointed sister. She was trained at Birmingham Infirmary, where she has since done sister's duty. She has also been charge nurse at the Military Families' Hospital. She holds the certificate of the Central Midwives Board.

Rochdale Infirmary.—Miss Emily Kate Luke has been appointed sister. She was trained at Manchester Southern Hospital. She has since been staff nurse at the Children's Hospital, Pendlebury, and has also been on the private nursing staff of the same institution. Subsequently she has been sister at Ancoats Hospital, Manchester.

Royal National Hospital for Consumption for Ireland, Newcastle, County Wicklow.—Miss J. Gertrude Powell has been appointed matron. She was trained at Sir Patrick Dun's Hospital, Dublin, and has since been matron of the Small-pox Auxiliary Hospital, Dublin, matron of the Royal National Hospital for Consumption, Newcastle (to which she now returns), matron of the Mercers' Hospital, Oulton, and superintending sister of the Royal Arsenal Hospital, Woolwich.

St. Pancras Infirmary (South), Cook's Terrace, Pancras Road, N.W.—Miss Gertrude Morris has been appointed sister. She was trained at St. Marylebone Infirmary. She has since been staff nurse at the Royal Sea Bathing Hospital, Margate.

Worcester General Infirmary.—Miss Elizabeth Rogers has been appointed sister. She was trained at the General Infirmary, Chester.

### The Nurses' Saloon.

(By our Shopping Correspondent.)

### NOVELTIES AT GARROULD'S.

The Hospital Nurses' saloon at Messrs. Garrould's, 150 to 160 Edgware Road, well repays a visit. There is no need to go from department to department in search of various requisites—they are all to be found here in one place. As for the cloaks, the "Rosemary," with detachable cape, is always a prime favourite. It is made, in all colours, in serge at 28s. 6d. and in army cloth at 35s. 6d., and is waterproof. Then there is the "Cecil" coat, a very well-built garment with two pockets, a yoke, and bishop sleeves; this is made in serge for 29s. 6d. and in cloth for 37s. I must not forget the ever-useful plain circular cloak which can be obtained from 18s. 9d. All these can be made to order for the same prices, and as all such work is done on the premises orders are very promptly executed. A neat little bonnet is the "Angela" with Warwick trimming and only costs 10s. 9d. without a veil and 14s. 9d. with one. For the same price can be had the "Cecil" with a velvet bow, while the popular "Sister Dora" is 10s. 9d. There is also a plain straw bonnet trimmed with silk at 6s. 11d. and with velvet at 7s. 11d. By the way, Messrs. Garrould have a new veiling called "Virnot" at 2s. 11d., which, being of closer texture than gossamer wears a good deal better and possesses the great recommendation that it can be washed and ironed and made to look as good as new again. They have also a novelty in belts, which are made of a special width, 3½ inches. These deep belts give a very smart appearance to uniform dress and can be fastened with three bone studs, for which there are corresponding buttonholes. The belts are stiffened ready for use and cost 3½d. each. For the narrower belts there are handy fasteners made of two studs on one foundation, thus greatly obviating the risk of losing these elusive little articles. The fasteners are silver-plated and cost 3½d. A novelty in caps is the "Annabel" for private nurses, which is both hygienic and pretty, as the fall of muslin at the back completely covers the hair and has a very graceful look. Made in plain muslin with strings they cost 2s. 6d. each and in spotted muslin 3s. 6d. There is a large variety of strings, all hand-made in Ireland, some of them being very daintly embroidered. There is a very serviceable-looking ward shoe composed of morocco at 5s. 11d., which has a flat heel with rubber underneath. Provision is also made for such feet as rebel against a flat heel and yet must not be indulged in an ordinary high heel. Shoes for these feet can be had with extra raised insteps which afford considerable relief to tired muscles. The heels are flat and covered with rubber; they are made in glacé at 8s. 11d. A shoe with an ordinary wooden heel, rubber underneath, can be had at 5s. 6d. Felt slippers with a double sole, half string and half felt, are both comfortable and durable and only cost 3s. 3d. With string soles only they are 2s. 9d. Very pretty pincushions at 6d. and 9d. are of red leather and silk in the shape of a "red cross." Messrs. Garrould make a speciality of hospital furniture, and they have a very ingenious operating table and ambulance combined, which can be employed for either purpose and costs only £12. This is found very useful in cottage hospitals. A special bed with pulley is raised extra high, thus saving the nurse from a good deal of stooping and making sweeping operations beneath it very easy; its price is £2 10s. T. Aubyn-Farrer's case-book for midwives at 9d. is proving a great success, and so is a receipt-book with counterfoil for private nurses.

### A Book and its Story.

### THE HOSPITAL MATRON IN FICTION.*

Ellis Deane's new book presents, like a former one, "A Raw Probationer," a lively picture of hospital life. In the former book the scene was laid in a large provincial hospital in England. In the present she carries her readers into far other surroundings, and shows the interior of a Colonial hospital in North Queensland. Bessie Deniston, the heroine, is a good delineation of an intelligent, conscientious young woman fresh from England struggling valiantly against the difficulties which confront her from the outset, when she arrives to take up the position of matron to the Tarraville Hospital. If the very elementary condition of things exists as described, the new country is lamentably behind the old in all matters connected with hospital *régime*, and the perusal of the experiences of the new matron, fresh from the Old Country, will be read with sympathy and interest by fellow-workers at home. It may perhaps act as a deterrent to those who, like the heroine, are thinking of finding in the Colonies higher salaries and more chances of promotion than are offered at home.

Bessie Deniston's first experience on arriving in Australia was the reverse of cheering. She had taken the post of nurse at the hospital at Mount Gambien, with a salary of £50 attached to it. Less than a month's experience proved the fallacy of the hope that in the new country opportunities would occur for putting into practice all that was best in her training. In this institution, at least, it was not possible. "It was a dead and alive sort of place . . . and it boasted at this time—1890—no method or management whatever. There was no matron, and the two day nurses—hours from 7 A.M. to 9 P.M.—had a sort of indefinite understanding that they were to look after the forty odd patients conjointly, and in the same manner superintend the domestic arrangements in general. The result was a confused and unsatisfactory one." An advertisement, attractively worded, had caught Bessie Deniston's eye one day in a Sydney paper, of necessity some days old before reaching Mount Gambien. A matron was required for the hospital at Tarraville, a large township in Northern Queensland. She decided at once to forward her testimonials and apply for the post. After some delay in the transmission of a telegram announcing her appointment she starts at once for Tarraville.

The circumstances which had led to the resignation of the late matron arose from the fact that the doctor and matron were diametrically opposed in their methods. "The doctor's bent was to be prodigally extravagant, the matron's to be uncompromisingly parsimonius. The Committee had found no fault with the doctor's mode of administration until the doctor and the matron openly quarrelled. Then in the inquiries and disclosures that ensued the Committee's eyes were opened (by the matron), and it rose to a man to condemn extravagances it ought to have condemned long ago, but had not taken the trouble to see. The end of the meeting was that the matron . . . had proffered her resignation . . . and the doctor, in high dudgeon . . . packed his belongings and left the hospital at a day's notice."

"Tarraville, though boasting some six medical men, was so incredibly behind the times in the administration of its hospital affairs that it had declined to allow any of its medical men to visit the hospital. Consequently, not one of them would ever go, in a case of necessity, without a heavy fee . . . no light consideration where there was an average of eighty patients with scarcely a chronic among them." Left without a doctor, the Committee hurriedly appoint another to fill his place. There was little time to make the choice, and Dr. Barratt, who was a young man staying in the town during an interval of travelling with an insurance agent, was chosen after coming forward himself without waiting for the vacancy to be advertised. He intended to ingratiate himself with the Committee and succeeded, in spite of a few facts which were not to his credit, and of which they were ignorant; the more important one being, that "there were doubts of his ability to produce a *bona fide* diploma." Installed at the hospital, he and the outgoing matron—still in possession—play into each other's hands with the hope that it may yet be possible for her to be re-elected, should the new matron not remain. This is the state of affairs when, after a fortnight's passage, Bessie Deniston arrives at Tarraville and drives at once to the hospital. "The cab had stopped in the light of a lamp swinging from the beam of a long verandah that brought into relief a stone-coloured line of wooden buildings out of the rainy blackness." The matron came forward to meet her; "with a smile on her narrow face that had nothing pleasant about it, but struck one as intended to hide a deeper resentment behind." They go into the matron's room, which appeared to Bessie Deniston, accustomed to the comfort of a matron's room at home, as being bare and comfortless. "It contained nothing beyond the barest necessities—a table, a few chairs, an uninviting horsehair couch. Nothing on the wooden floor, nor any ornament of any sort anywhere; not so much as a fire, though it was mid-winter and very chilly: even for those whose blood had not been thinned by long seasons of tropical heat—the gas burned dimly and gave but an uncertain light." The following day, having been curtly told by the late matron that she could go where she liked, and do as she pleased, refusing, at the same time, to initiate her into the routine of the day's duties or to give her any information connected with her post, she walks out to inspect the exterior of the hospital. "The institution had quite a number of small detachments. The main block was a large square building of two stories, with a belt of balcony above, and a terrace or verandah below, running out from wide portals into well-kept garden grounds. Nowhere else were there two stories, but every one-storied building had these spacious verandahs and garden allotments. . . It was late July, and, therefore, Australia's mid-winter. . . The whole earth was dew-washed and many flowers abloom. The trees, the grass, the trailing creepers garlanding the verandah posts, were green with a green that they know not in a tropical summer time; and away in the distance was the sparkling sea, and the heart of the Englishwoman went out to it all. How favoured she had been by Providence, she told herself, to have been entrusted with the care of such a place! Unfortunately, the new matron had not seen the interior of her new domain. But what she saw through an open glass door disclosed in one of the wards such a scene of disorder and discomfort that her heart sank at the sight of it. Further incursions disclosed a total disregard of sanitary regulations. The struggle of the new matron to transform the interior working of Tarraville Hospital from the state of disorder which prevailed in every department, into one nearly allied to a well-managed institution at home; and her patient, self-sacrificing persistence, in spite of covert opposition from the doctor in charge, should be read by everyone interested in her position, which is one, apparently, common enough in provincial hospitals in the Colonies.

* "The New Matron." By Ellis Deane. (Digby, Long and Co. 6s.)

### Notes and Queries.

**REGULATIONS.**

**The Editor is always willing to answer in his olumn, without any fee, all reasonable questions, as soon as possible.**

**But the following rules must be carefully observed.**

**1. Every communication must be accompanied by the name and address of the writer.**

**2. The question must always bear upon nursing, directly or indirectly.**

**If an answer is required by letter a fee of half-a-crown must be enclosed with the note containing the inquiry.**

*Training.*

(40) Is it likely that I and a friend can receive training together? What is the earliest age required, and should we start at an infirmary?—*Simple.*

You would probably find it easier to get into a Poor-law hospital together, but it must be a large one. Twenty-three is the usual age, but some institutions will take you a little earlier.

*School Matron.*

(41) Will you tell me the best nursing manual for a school (girls') matron?—*Constant Reader.*

If you require a simple and practical elementary book there is none better than "Lectures on Home Nursing for the Poor"; it is full of practical hints; price 1s. 8d. post free. "Fever Nursing," by William Hardy, M.D., 1s. 1d. post free, might be of use; and "Nursing: its Theory and Practice," price 3s. 10d. post free. All these can be had from the Scientific Press, 28 Southampton Street, Strand, W.C.

*Agreement with Nursing Homes.*

(42) If a nurse signs an agreement not to nurse near a nursing home she joins, can she, if discharged for not being strong, break the agreement and pratise near the home?—*Inquirer.*

Certainly not. The nurse should not have signed the agreement if she did not mean to keep it.

*Training School.*

(43) Can you advise me as to whether I should join a Union Infirmary to train as a nurse, or a general hospital?—*Ignoramus.*

If you join a general hospital, and still fancy Poor-law nursing, you can secure a post at an infirmary afterwards. Therefore if you do not wish to continue in the Poor Law service, train at a general hospital first.

*Uniform.*

(44) Are children's nurses allowed to wear a long veil at the back of their bonnets?—*Brixton.*

There is no law to prevent it. We have seen such, but it is bad taste, as sailing under false colours.

*Oxygen.*

(45) How is oxygen administered; through water or otherwise?—*Mona.*

If you are required to administer oxygen you should ask the medical man to instruct you.

*Indian Nursing Service.*

(46) What steps must one take to join the Indian Nursing Service, military or civil?—*Abingdon.*

Write to the Countess of Minto, Vice-Regal Lodge, Simla, India, as to civil nursing, and to Queen Alexandra's Imperial Military Nursing Service for India, the India Office, St. James's Park, S.W., as to military nursing.

*Harrogate.*

(47) Can you tell me the addresses of private nursing homes in Harrogate?—*Biggleswade.*

We do not give the addresses of private homes in these columns. There is a little book which contains particulars of nursing homes. Title, "Medical Homes for Private Patients." The Scientific Press, 28 Southampton Street, Strand, will send it, price 7d. post free.

**Handbooks for Nurses.**

| | Post Free. |
|---|---|
| "How to Become a Nurse: How and Where to Train." | 2s. 4d. |
| "Nursing: its Theory and Practice." (Lewis.) ... | 3s. 6d. |
| "Nurses' Pronouncing Dictionary of Medical Terms." | 2s. 6d. |
| "Complete Handbook of Midwifery." (Watson.) ... | 6s. 4d. |
| "Preparation for Operation in Private Houses." ... | 0s. 6d. |

Of all booksellers or of The Scientific Press, Limited, 28 & 29 Southampton Street, Strand, London, W.C.

### For Reading to the Sick.

THE BLESSINGS OF SORROW.

Sustain me, Lord, and let me neither shrink
Nor scorn the rod of painful discipline,
The cup my Father gives me, I would drink,
And bend my will submissively to Thine.

I know the cross is needful, and I know
In love, and not in wrath, Thou chastenest;
The sufferings Thy children undergo
But fit them sooner for eternal rest.

*From the Dove on the Cross.*

It may seem strange to some to speak of the blessings of sorrow. We would say at first thought, "Surely nothing good can come from anything so terrible." Yet the Word of God assures us, and the experience of the ages confirms the assurance that many of the richest and best blessings of life come out of affliction.

We do not know what we owe to our sorrows. Without them we should miss the sweetest joys, the divinest revealings, the deepest experiences of life. Afflictions are opportunities. They come to us bearing gifts. If we can accept them they leave in our hand heavenly treasures. Not to be able to receive the bearer of the blessings is to miss the blessings and to be poorer all the rest of our days.

One of the most striking visions of heaven granted to the revelator on Patmos was that of a glorified company who seemed to surpass all the other blessed ones in the splendour of their garments and the radiant honour of their state. They were arrayed in white robes, carried palms in their hands, and stood nearest the Throne and the Lamb. We would have said that these were the children of joy, that they had come up from earth's scenes of gladness, that their condition in life had been one of exceptional ease and freedom from trouble, that they had never known a care or a grief. But when the question was asked, "These which are arrayed in the white robes, who are they, and whence came they?" the answer was, "These are they which come out of the great tribulation." They were the children of earth's sorrow.

This vision would seem to teach us that those redeemed ones who on earth have had the most affliction in heaven attain the highest honour. . . . They are nearest to Christ, too, among the glorified, verifying the promise that they who suffer with Him shall also reign with Him."—*Dr. J. R. Miller.*

There is no death in heaven;
For they who gain that shore
Have won their immortality,
And "they can die no more."

There is no death in heaven;
But when the Christian dies,
Made thus co-heir with angels,
They waft him to the skies.

*G. S. De M. Rutherford.*

